# Saphenous vein valve assessment utilizing upright CT to potentially improve graft assessment for bypass surgery

**DOI:** 10.1038/s41598-021-90998-7

**Published:** 2021-06-02

**Authors:** Takehiro Nakahara, Minoru Yamada, Yoichi Yokoyama, Yoshitake Yamada, Keiichi Narita, Nobuaki Imanishi, Masataka Yamazaki, Hideyuki Shimizu, Jagat Narula, Masahiro Jinzaki

**Affiliations:** 1 Department of Radiology, Keio University School of Medicine, Shinanomachi 35, Shinjyuku, Tokyo, 160-8582 Japan; 2 Department of Plastic and Reconstructive Surgery, Keio University School of Medicine, Tokyo, Japan; 3 Department of Cardiovascular Surgery, Keio University School of Medicine, Tokyo, Japan; 4 Mount Sinai Heart, Icahn School of Medicine at Mount Sinai, New York, NY USA

**Keywords:** Cardiology, Medical imaging, Radiography, Tomography

## Abstract

Saphenous veins (SVs) are frequently employed as bypass grafts. The SV graft failure is predominantly seen at the valve site. Avoiding valves during vein harvest would help reduce graft failure. We endeavored to detect SV valves, tributaries, and vessel size employing upright computed tomography (CT) for the raw cadaver venous samples and in healthy volunteers. Five cadaver legs were scanned. Anatomical analysis showed 3.0 (IQR: 2.0–3.0) valves and 13.50 (IQR: 10.00–16.25) tributaries. The upright CT completely detected, compared to 2.0 (IQR: 1.5–2.5, *p* = 0.06) valves and 9.5 (IQR: 7.5–13.0, *p* = 0.13) tributaries by supine CT. From a total of 190 volunteers, 138 (men:75, women:63) were included. The number of valves from the SF junction to 35 cm were significantly higher in upright CT than in supine CT bilaterally [upright vs. supine, Right: 4 (IQR: 3–5) vs. 2 (IQR:1–2), *p* < 0.0001, Left: 4 (IQR: 3–5) vs. 2 (IQR: 1–2), *p* < 0.0001]. The number of tributaries and vessel areas per leg were also higher for upright compared with supine CT. Upright CT enables non-invasive detection of SV valves, tributaries, and vessel size. Although not tested here, it is expected that upright CT may potentially improve graft assessment for bypass surgery.

## Introduction

For decades, the saphenous vein (SV) has served as the most reliable bypass conduits for coronary and peripheral artery revascularization. Over 250,000 coronary artery bypass grafts and over 80,000 lower extremity vein grafts are performed annually in the United States alone^1^. The average rate of CABG has been reported to be 44 per 100,000 individuals^2^, and almost 80% of bypass grafts include saphenous veins (SVs)^2,3^. However, SV graft failure during follow-up is not uncommon^3,4^, wherein most stenotic lesions are focal and often occur either at the valve sites or anastomotic regions^5–7^. Therefore, it has been proposed that non-inclusion of venous samples with valve sites may reduce SV graft failure. This concept has led to the development of valvulotomy^8^; however, this approach did not improve prognosis^9^.

**Figure 1:**
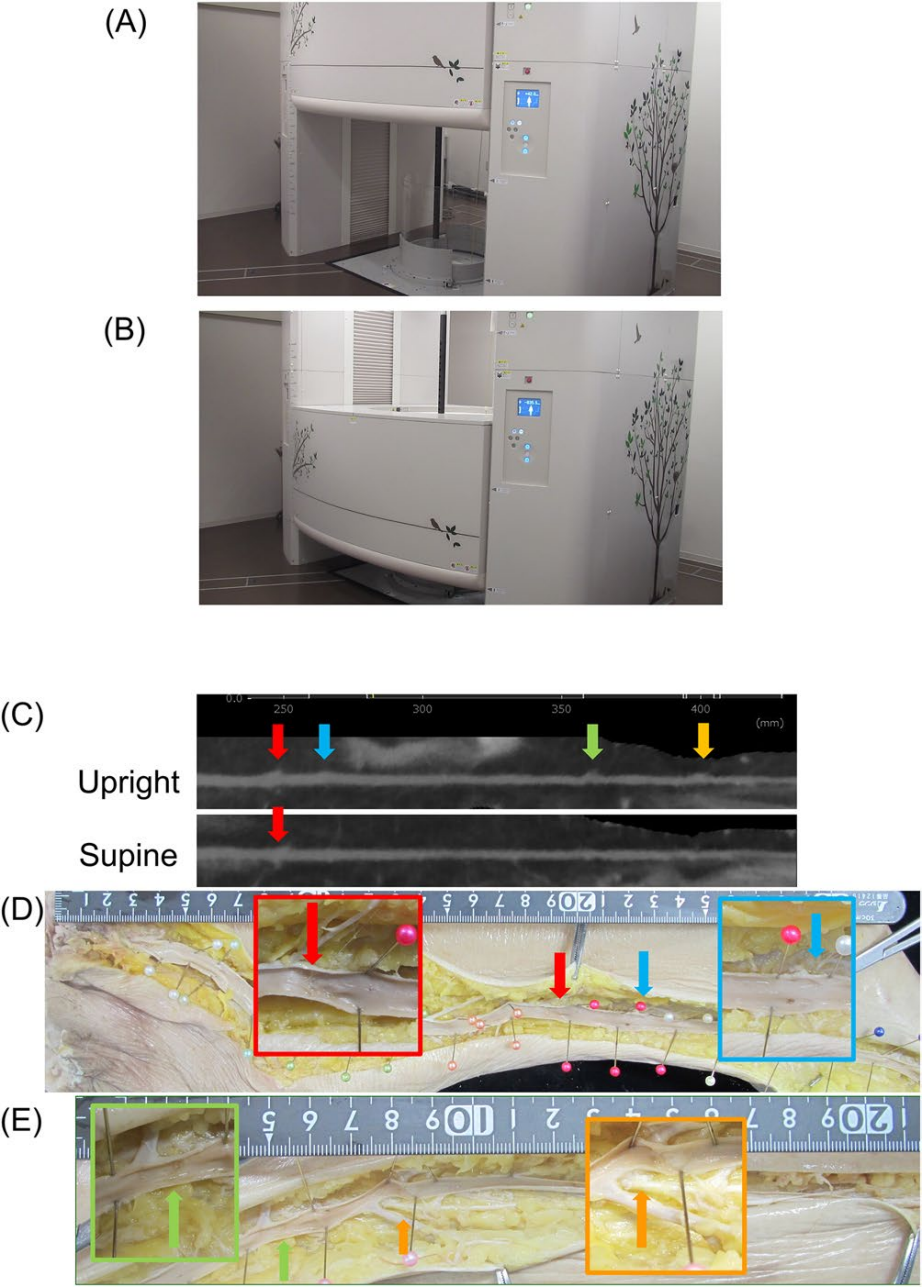
An overview of upright CT and CT images of raw cadaver lower extremities and anatomical analyses after being fixed with formaldehyde. (**A**) Gantry in the up position. (**B**) Gantry in the down position. Subjects stand in the center of the gantry and the gantry moves up to down to scan. (**C**) MPR images of SV based on both upright CT and supine CT. Four valves (arrows) were identified in upright CT, however; some of them had difficulty to identify in supine CT. (**D** and **E**) The upper part (**D**) and lower part (**E**) of the same lower extremity. After image acquisitions, the specimen samples were fixed by formaldehyde and saphenous vein was dissected and made a long single incision along the venous path from the cannulation point to the SF junction. Four valves were identified in anatomical analysis (arrows. the color of the arrows was corresponded in the CT images in panel C) and confirmed the results of upright CT analysis. The tributaries were also analyzed in the same manner.

The valves of venous systems with venous muscle pumps allow the blood to return to the heart against gravity and generally demonstrate expansion at the valve site during in the upright position ^10^. We have developed an upright CT (Fig. 1A,B) to negate the postural effect of gravity^11^ and render the clinical imaging as close to the physiological state as possible ^12,13^. Employing the upright CT, we aimed to detect SV valves, tributaries, and venous areas and compare them with those obtained using standard supine CT imaging.

## Materials and methods

### Cadaver study

Cadaveric specimens were obtained from the Willed Body Program (a whole-body donation program which written informed consent was obtained from doner or next of kin at Department of Anatomy, Keio University School of Medicine) and were investigated in accordance with institutional regulations. Lower extremities, with no scars and no signs of vascular disease, were obtained from raw cadavers. Lower extremities were disarticulated at the hip joint and the external iliac artery, external iliac vein, and the surrounding soft tissue remaining attached to the specimens. The distal position of saphenous vein was cannulated with a 22-Gauze catheter at above ankle level, and phosphate buffered saline (PBS) with 10% polyethylene glycol ^14^ was infused under continuous natural dripping by gravity during the scan to reproduce the venous reflex. The CT images were acquired as ex-vivo imaging of the disarticulated leg. Thereafter, the leg specimens were fixed in formaldehyde and the saphenous vein was dissected and made a long single incision along the venous path from the cannulation point to the saphenous-femoral junction (SF junction) (Supplemental Fig. 1). The SV valves and tributaries positions were macroscopically identified as the reference standard for CT images.

### Volunteer study

This volunteer prospective study was approved by the Institutional Review Board (Keio University Independent Ethics Committee) (Clinical Trial number: UMIN000026586) and written informed consent was obtained from all participants. Healthy volunteers were recruited from a volunteer recruitment company from July 2017 to March 2019; volunteers of over 30 years of age were requested so that they could understand the purpose of the study. Individuals with a history of hypertension, dyslipidemia, diabetes, smoking, or those who had previously undergone cardiac surgery or were currently receiving treatment were excluded.

### Image acquisition

A conventional 320-detector row CT (Aquilion ONE, Canon Medical Systems Corporation, Otawara, Japan) was prospectively performed for all volunteers in the supine position. In addition, and upright CT (prototype TSX-401R, Canon Medical Systems Corporation, Otawara, Japan) ^11^ was performed in the standing position immediately after the conventional procedure. The upright and conventional supine CT scanners were placed next to each other, and the two examinations were consecutively performed. The upright CT system is characterized by up-and-down movements of a transverse 320 row-detector gantry (isotropic 0.5 mm in detector size), with a bore size of 780 mm, a gantry rotation speed of 0.275 s, a maximum vertical speed of 100 mm/s, and a 1200 view at optimal performance. Scanning was performed at 100kVp and at a gantry rotation speed of 0.5 s in the helical scan mode (80-row detector), with a noise index of 24 and helical pitch of 0.8 for the body trunk from the level of superior margin of the external acoustic meatus to the lowest position of the upright CT (37 cm height).

For cadaver study, cadaver legs were placed in plastic moulds and whole samples were scanned in both supine and upright CT scanners. The plastic box containing the sample was fixed on a pedestal (42 cm height) and the tube current for scanning was set at approximately 600 mA to compensate for the noise from the fixtures.

Image reconstruction was performed using Adaptive Iterative Dose Reduction 3D (Canon Medical Systems Corporation, Otawara, Japan), which could reduce the radiation dose.

### Image analysis

CT images were transferred to an off-line workstation (SYNAPSE VINCENT, FujiFilm, Tokyo, Japan) and multiplanar reconstruction (MPR) images were developed from the SF junction of the saphenous vein both for cadaveric images and clinical images. The positions and number of valves were counted visually. Simultaneously, the vessel area in cross-sectional images were measured to calculate the dilation ratios (vessel area at valve /vessel area at reference segments). The reference segments were set at the average of the vessel area of the proximal and distal positions of the valves. The dilation ratio was derived in the cadaveric study and applied for the volunteer study to study the valves. The number of tributaries were counted visually. The vessel area was measured of the cross-section image on the 15 cm from the SF junction. For the cadaveric study, two observers analyzed the data with a consensus reading. For the volunteer study, all measurements were performed in a blinded and randomized manner.

### Statistical analysis

Intra-observer and inter-observer variability for the assessment of valves, tributaries, and cross-sectional measurements were examined using Bland–Altman analysis and Pearson’s correlation. Data were presented as median (interquartile range [IQR]; i.e., 25th to 75th percentile, or Q1, Q3). Continuous data were compared using a Wilcoxon rank-sum test between the 2 groups. A Spearman Rank Correction coefficient test was used for the assessment of linear correlation of 2 parameters. A 2-sided *p* value of < 0.05 was considered statistically significant. All analyses were performed using SAS software, version 9.4. (SAS Institute Inc., Cary, North Carolina).

## Results

### Cadaver study

Five legs were dismembered from cadavers (age range 78–93 years, mean 88.4 ± 6.0 years, 4 left and 1 right leg, 4 female and 1 male). On visual assessment 3.0 (IQR 2.0–3.0) valves and 13.50 (IQR 10.00–16.25) tributaries per leg were observed in the SV (Fig. 1C–E).

The upright CT images accurately identified the valves and tributaries, while the identification was difficult using conventional supine CT. The upright CT image showed 3.0 (IQR 2.0–3.0) valves and 13.50 (IQR 10.00–16.25) tributaries, while supine CT images showed 2.0 (IQR 1.5–2.5, *p* = 0.06) valves and 9.5 (IQR 7.5–13.0, *p* = 0.13) tributaries per leg; given a small sample size the difference however was not statistically significant. The expansive venous diameter at the valve site (referred to as dilation ratio) in the upright CT images was 1.56 (IQR 1.44–1.83, range 1.20–4.24), and the minimum dilation ratio of “1.20” was used to confirm the valve site in the volunteer images.

### Volunteer study

Of the 190 volunteers, 38 who received the scan with 120 kV were excluded; another 14 volunteers were excluded where complete CT data from the SF junction to 35 cm could not be obtained due to their short statures. We report the CT characteristics of the SV from 138 volunteers (men 75, women 63) who completed the study (Table 1).

**Table 1:** Volunteer characteristics (n = 138).

N	138	(Male:75, Female:63)
Age (years old)	46.00	(IQR: 37–53, range: 30–80)
Body height (cm)	165.0	(IQR: 158.8–171.0)
Body weight (kg)	62.0	(IQR: 52.5–69.3)
BMI (kg/m^2^)	22.3	(IQR: 20.4–24.1)

**Figure 2:**
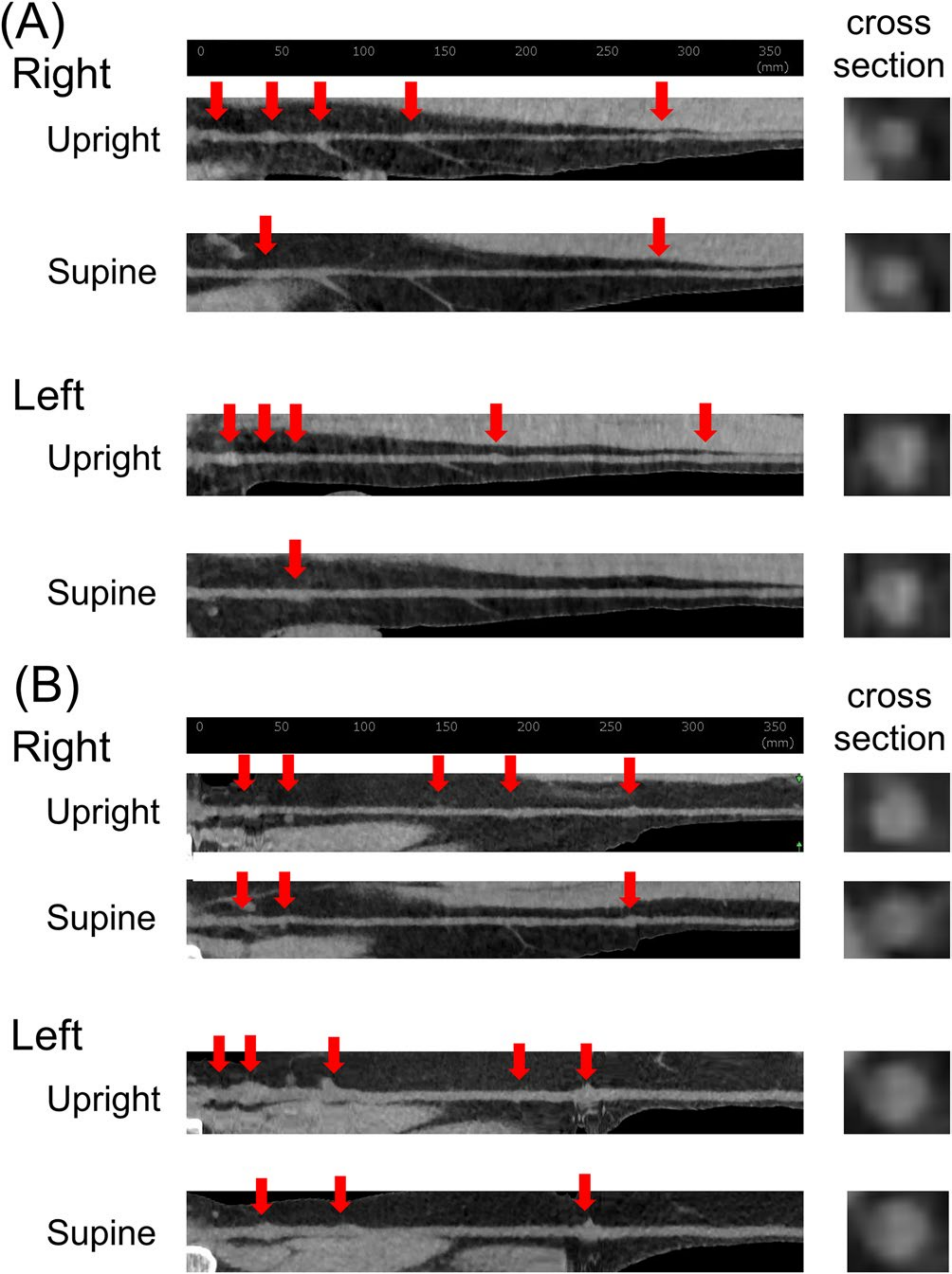
A representative case from the male and female volunteers. (**A**) A 39-year-old male (B.H. 181.3 cm, B.W. 72.1 kg, BMI 21.9). Upright CT detects 5/5 valves (arrows) and 18/13 tributaries (right/left sides). The cross-sectional images on the 15 cm from the SF junction showed a size of 10/22 mm^2^ (right/left sides). Supine CT detects 2/1 valves (arrows) and 14/13 tributaries (right/left sides). The cross-sectional images on the 15 cm from the SF junction showed a size of 10 /15 mm^2^ (right/left sides). (**B**) A 52-year-old female (B.H. 162.0 cm, B.W. 47.8 kg, BMI 18.2). Upright CT detects 5/5 valves (arrows) and 14/13 tributaries (right/left sides). The cross-sectional images on the 15 cm from a SF junction showed the size of 19/29 mm^2^ (right/left sides). Supine CT detects 3/3 valves (arrows) and 13/14 tributaries (right/left sides). The cross-sectional images on the 15 cm from the SF junction showed a size of 16/26 mm^2^ (right/left sides).

Firstly, 40 CT leg images from 10 consecutive volunteers taken from both sides and in both the supine and upright positions, were analyzed by two observers, with a high intra-/inter-observer agreement noted (Supplemental Figs. 2, 3 and 4). The upright images allowed for the identification of valves and tributaries (Fig. 2). It was difficult to characterize valves and tributaries in supine CT images, similar to that seen in cadaveric studies.

**Figure 3:**
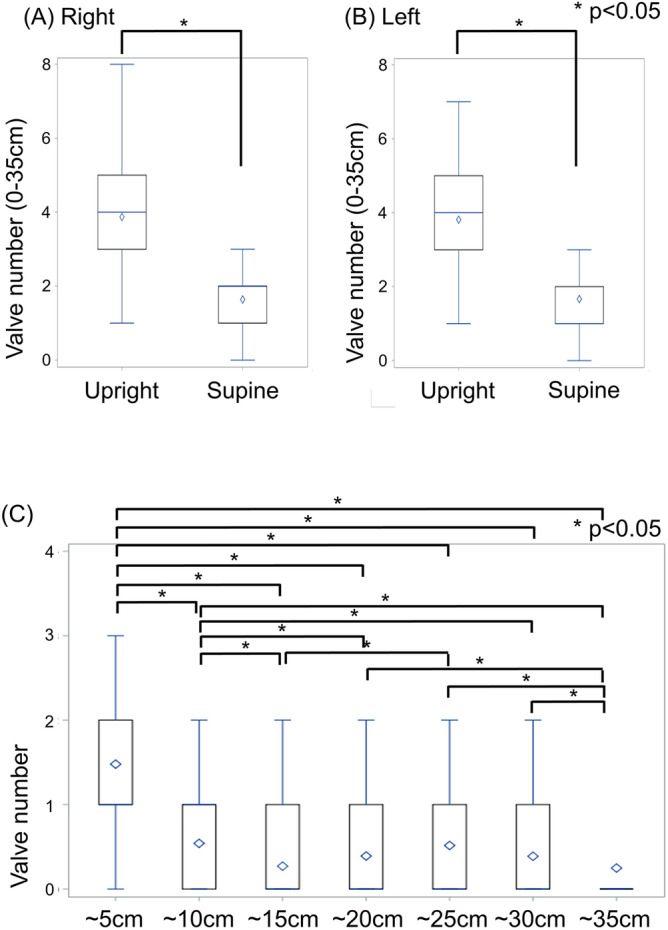
Valve numbers and distribution in upright and supine CT. Valve numbers in the upright and supine CT in (**A**) right side and (**B**) left side. On both sides, the valve numbers were significantly higher in the upright CT. (**C**) The distribution of valves in upright CT was highest in the lesion from the SF junction to 5 cm, followed by 5 to 10 cm.

The number of valves was significantly higher in upright CT than in supine CT on both sides [upright right side: 4 (IQR: 3–5) vs. supine right side: 2 (IQR: 1–2), *p* < 0.0001, upright left side: 4 (IQR: 3–5) vs. supine left side: 2 (IQR: 1–2), *p* < 0.0001] (Fig. 3A,B). The dilation ratio at the valve site was 1.85 (IQR 1.57–2.25). The frequency of valve distribution was maximum between 0 and 5 cm from the SF junction, followed by 5 to 10 cm from the SF junction (Fig. 3C).

**Figure 4:**
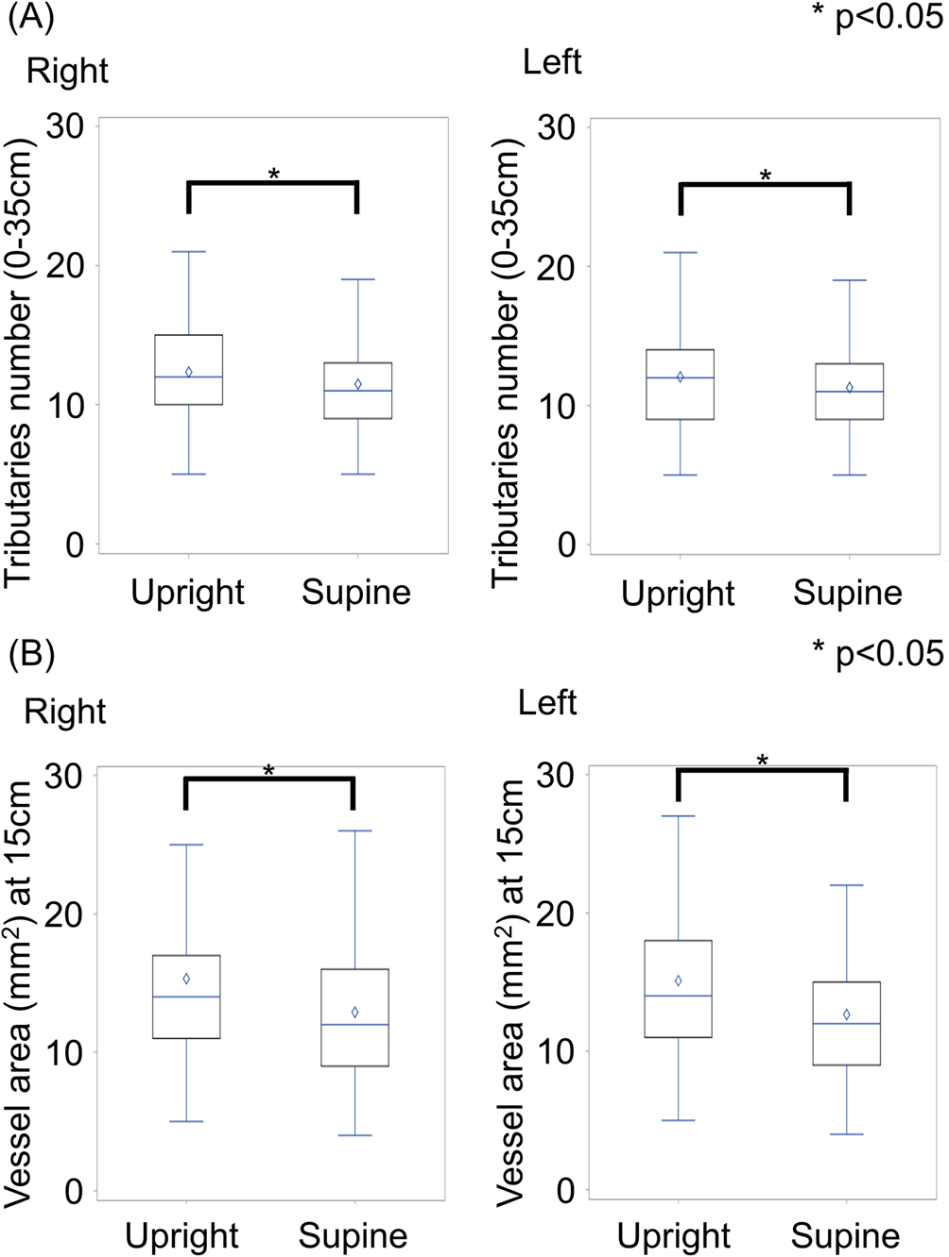
The number of tributaries and vessel areas in upright and supine CT. (**A**) The number of tributaries in upright and supine CT in right and left sides. In both sides, the number of tributaries was significantly higher in the upright CT. (**B**) The vessel area (15 cm from the SF junction) in upright and supine CT in right and left sides. In both sides, the vessel area was significantly higher in the upright CT.

The number of tributaries was also higher in the upright CT than in the supine CT [upright right: 12 (IQR: 10–15) vs. supine right: 11 (IQR: 9–13), *p* < 0.0001; upright left: 12 (IQR: 9–14) vs. supine left: 11 (IQR: 9–13), *p* < 0.0001] (Fig. 4A).

The area of the cross-sectional image at 15 cm from the SF junction was larger in the upright CT [upright right side: 14 (IQR: 11–17) mm^2^ vs. supine right: 12 (IQR: 9–16) mm^2^, *p* < 0.0001; upright left: 14 (IQR: 11–18) mm^2^ vs. supine left: 12 (IQR: 9–15) mm^2^, *p* < 0.0001] (Fig. 4B).

## Discussion

This study showed that the SV valves were clearly visualized in the standing position utilizing upright CT both in cadaveric legs and in-vivo study of healthy volunteers. In the cadaver study, SV valves and tributaries identified by upright CT were confirmed by anatomical analysis. A minimum dilation ratio of “1.20” was found to be the cut-off point to define the level of the valve to confirm the valve site in the in-vivo study. In the volunteer study, upright CT detected a larger number of valves and tributaries as well as a larger size of vessel area compared with supine CT. We propose that upright CT could have an advantage for SV characterization prior to bypass surgeries. We envision that this method can allow for SV valve and tributary assessment in order to locate the optimal part of the saphenous vein (minimum SV valves and tributaries with sufficient diameter) before harvesting the saphenous vein for bypass grafts, although further studies need to be done.

SV grafts have the advantages of having long lengths and being easily available while being more resistant to iatrogenic injury and less susceptible to vasospasm ^15^. They have considerably longer length compared to the other types of grafts. SV grafts have been most used for non-LAD coronary territories worldwide. It has the major disadvantage of a high graft failure rate^2,3^, with approximately 40–50% of grafts occluding at 10 years after CABG surgery, of which 10–25% are occluded within the first year post-operatively ^4,16,17^. In comparison, the occlusion rates of other grafts are lower, for instance the radial artery shows a failure rate of 17–37% ^16,18^. One of the causes of SV failure is the endothelium dysfunction during the harvest, new harvesting techniques being employed reduce dysfunction and graft failure, such as including bridge, no-touch, endoscopic resections^3^. Another possible case is the presence of venous valves. Although the mechanisms of primary stenosis of coronary artery bypass grafts and peripheral artery bypass grafts have been suggested to be different, they share thrombosis as the basis of luminal loss ^19^. In SV grafts, valve sites frequently gather clots ^20^, which cause valve dysfunction, scarring and develop post-thrombotic syndrome ^19^; the response of local smooth muscle cells to injury is found to be accelerated in-vitro ^21^. Clinical studies have confirmed that valve sites frequently cause stenotic lesions when SV graft reoperations are performed ^5–7,22–24^. We assumed that knowing the SV valve position and avoiding the valve sites during harvesting may offer another approach to reduce SV graft failure.

To identify the best portion of the SV as a conduit for bypass surgery, the number and position of valves in the SV were reviewed. A Brazilian study characterized 60 veins from 30 adult cadavers and reported that the average number of valves from the medial epicondyle of the femur to the saphenous hiatus were 4.77 and 4.87 on the left and right sides, respectively ^25^. Similarly, a Japanese study of 26 SVs from 20 adult cadavers found 111 valves (average: 4.27) between the SF junction and the upper patellar margin compared to 63 (average: 2.42) valves between the upper patellar margin and the medial malleolus ^26^; they also reported that greatest number of valves were observed within 10 cm from the SF junction and between 35 and 45 cm from the SF junction. Because we identified 4 (IQR: 3–5) valves from 0 to 35 cm from the SF junction and the distribution of valves was mainly observed between 0 and 10 cm, the results of our volunteer study were comparable with these autopsy studies; however, the data beyond 35 cm from the SF junction were not available in this study. Therefore, upright CT was able to accurately characterize of SV valves, which could not be easily done using conventional computed tomography.

Another advantage of upright CT is its noninvasive identification of tributaries and vessel areas. Vessel size is an important factor to predict graft failure and a luminal diameter of over 2.0 mm is preferable for SV graft patency and longevity ^3,16,27^. New harvesting techniques with less invasiveness to the SV may reduce damage compared with conventional open harvesting techniques, however; it is difficult to evaluate the valve sites and vessel sizes in the whole SV. In addition, it is important to be able to ligate all side tributaries ^28^. Upright CT allows to locate valves, identify tributaries, and accurate measurement vessel sizes in non-invasive manner, and should be incorporated in the intraoperative strategy before performing graft harvesting.

Recently upright (or weight-bearing) CT ^29^ and MRI ^30^ have been proposed to evaluate the effect of gravity. However, previous upright CT scanners were equipped with a cone beam CT, and their scan range was limited. Upright MRI has a lower spatial resolution than CT ^31^. Compared to these machines, the upright CT used in the current study has a wide-scan range with quick motion and high spatial resolution, which is similar to a high-end supine CT in the standing position. It has an ability to evaluate blood distribution of whole body ^11^, and may contribute to the development of *phlebology,* which has more room for the development compared with *arteriology.*

### Limitations of the study

Although a novel attempt, this study has several limitations. First, because the upright CT used was a prototype machine, this is a single center study. Second, the protocol of this study was designed to obtain body-trunk images. Thus, the scan range of this prototype machine could not be detected beyond a height of 37 cm, which did not allow for imaging of the entire saphenous vein. However, we could acquire the entire SV with a pedestal to reach a height of 42 cm, which we utilized for the evaluation of the ankles ^32^. Third, valve insertion sites were identified by defining the venous (expansive) dilation ratio compared to the lumen, with a cut-off point of 1.20 used based on anatomic observation in the cadaveric limb. The application of a workstation is being developed to enable to automatic detection of the valve in future clinical studies. Fourth, we could not identify the valve leaflet directly, because the upright CT scans were performed without contrast medium and the limit of the spatial resolution of CT is 0.4 to 0.6 mm. Invasive OCT with 15 micro-meter spatial resolution ^31^ has allowed for the recognition of culprit valve lesions after CABG ^33^. However, it is difficult to identify SV valves in supine CT, even with the use of contrast materials. Thus, this approach, which utilized the difference in venous pressure upon changes in posture^34^, may have practical use for detecting valves positions.

## Conclusions

Upright CT enables detection of SV valve sites, tributaries, and vessel sizes noninvasively. It may potentially assist the strategies for SV harvesting to improve patency of bypass-grafts.

## Supplementary Information

Supplementary Figures.

## Data Availability

The datasets generated during and/or analyzed during the current study are available from the corresponding author upon reasonable request.
