# Supplementary material for: Saphenous vein valve assessment utilizing upright CT to potentially improve graft assessment for bypass surgery
Source: Sci Rep. 2021 Jun 2;11:11602. doi: 10.1038/s41598-021-90998-7 (PMC8172633; doi:10.1038/s41598-021-90998-7)

Saphenous vein valve assessment utilizing upright CT to potentially improve graft assessment for bypass surgery.

Takehiro Nakahara, MD, PhD, [1] Minoru Yamada, PhD, [1] Yoichi Yokoyama, MD, [1] Yoshitake Yamada, MD, PhD, [1] Keiichi Narita, MD, PhD, [1] Nobuaki Imanishi, MD, PhD, [2] Masataka Yamazaki, MD, PhD, [3] Hideyuki Shimizu, MD, PhD, [3] Jagat Narula, MD, PhD, [4] and Masahiro Jinzaki, MD, PhD, [1]

1. Department of Radiology, Keio University School of Medicine, Tokyo, Japan

2. Department of Plastic and Reconstructive Surgery, Keio University School of Medicine, Tokyo, Japan

3. Department of Cardiovascular Surgery, Keio University School of Medicine, Tokyo, Japan

4. Mount Sinai Heart, Icahn School of Medicine at Mount Sinai, New York, New York

Address for Correspondence: Masahiro Jinzaki,

Departments of Radiology, Keio University School of Medicine, Shinanomachi 35, Shinjyuku,Tokyo,160-8582, Japan, Tel:+81-3-5363-3837. Fax: +81-3-3353-1977, e-mail: jinzaki@rad.med.keio.ac.jp

Short title: Saphenous vein assessment utilizing upright CT.

Supplemental Figure legends

Supplemental Figure.1 An overview of cadaver lower extremities.

Fresh cadaver lower extremities, with no scars and no signs of vascular disease, were selected and harvested from bodies of individuals. All lower extremities were disarticulated at the hip joint and the external iliac artery, common iliac vein and the surrounding soft tissue remained attached to the specimens. The distal position of saphenous vein was cannulated with 22-Gauze catheter above ankle level (A, arrow) and phosphate buffered saline (PBS) with 10% polyethylene glycol was insulted during the scan to reproduce the venous reflex. After image acquisitions, formaldehyde was injected via the catheter and soaked with formaldehyde thee overnight. The saphenous vein was dissected from the cannulation point and made a long single incision along the venous path from the cannulation point to the saphenous hiatus (B). Anatomically investigated the positions and the numbers of valves and tributaries as the reference standard for CT images.

Supplemental Figure.2 The reproducibility of valves assessment.

To evaluate the reproducibility of valves assessment, intra- and inter- observer viability is assessed by Bland-Altman analysis. The valve was assessed visually and confirm if the

valve area was 1.2 holds larger compared with the average vessel area at proximal and distal position of valves. (A, B). In intra observer viability, an observer (TN) assessed valves in total 40 legs from continuous 10 patients of both side with supine and upright positions twice 3 months apart. The Pearson's R shows high correlation (A: r=0.98, p<0.0001) and the Bland-Altman analysis shows agreement of intra observer reproducibility (B). (C, D) In inter observer viability. Two observers (TN and YY) assessed the same 40 legs patients independently. The Pearson's R shows high correlation (C: r=0.97, p<0.0001) and the Bland-Altman analysis shows excellent inter-observer agreement (D).

Supplemental Figure.3 The reproducibility of tributaries assessment.

To evaluate the reproducibility of tributaries assessment, intra- and inter- observer viability is assessed by Bland-Altman analysis. (A, B) Intra observer viability. An observer (TN) assessed tributaries in total 40 legs from continuous 10 patients of both side with supine and upright positions twice 3 months apart. The Pearson's R shows high correlation (A: r=0.97, p<0.0001) and the Bland-Altman analysis shows agreement of intra observer reproducibility (B). (C, D) Inter observer viability. Two observers (TN and YY) assessed the same 40 legs patients independently. The Pearson's R shows high

correlation (C: r=0.96, p<0.0001) and the Bland-Altman analysis shows excellent inter-observer agreement (D).

Supplemental Figure.4 The reproducibility of vessel area assessment at the 15 cm from the SF junction assessment.

To evaluate the reproducibility of vessel area at the 15 cm from the SF junction assessment, intra- and inter- observer viability is assessed by Bland-Altman analysis. (A, B) Intra observer viability. An observer (TN) assessed vessel area at the 15 cm from the SF junction in total 40 legs from continuous 10 patients of both side with supine and upright positions twice 3 months apart. The Pearson's R shows high correlation (A: r=0.97, p<0.0001) and the Bland-Altman analysis shows agreement of intra observer reproducibility (B). (C, D) Inter observer viability. Two observers (TN and YY) assessed the same 40 legs patients independently. The Pearson's R shows high correlation (C: r=0.91, p<0.0001) and the Bland-Altman analysis shows excellent inter-observer agreement (D).

Supplemental Figure.1

(A)

(B)

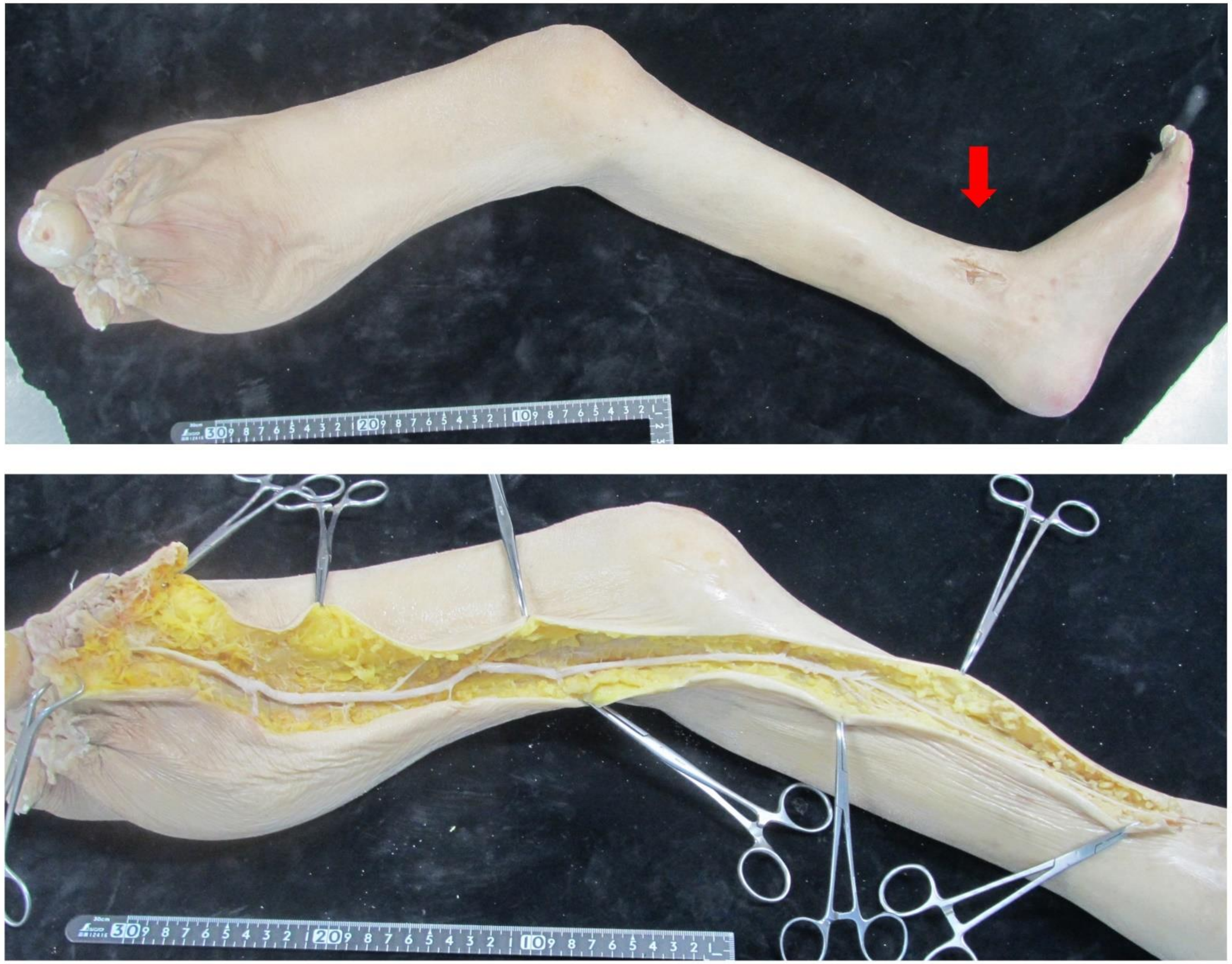

Supplemental Figure.2

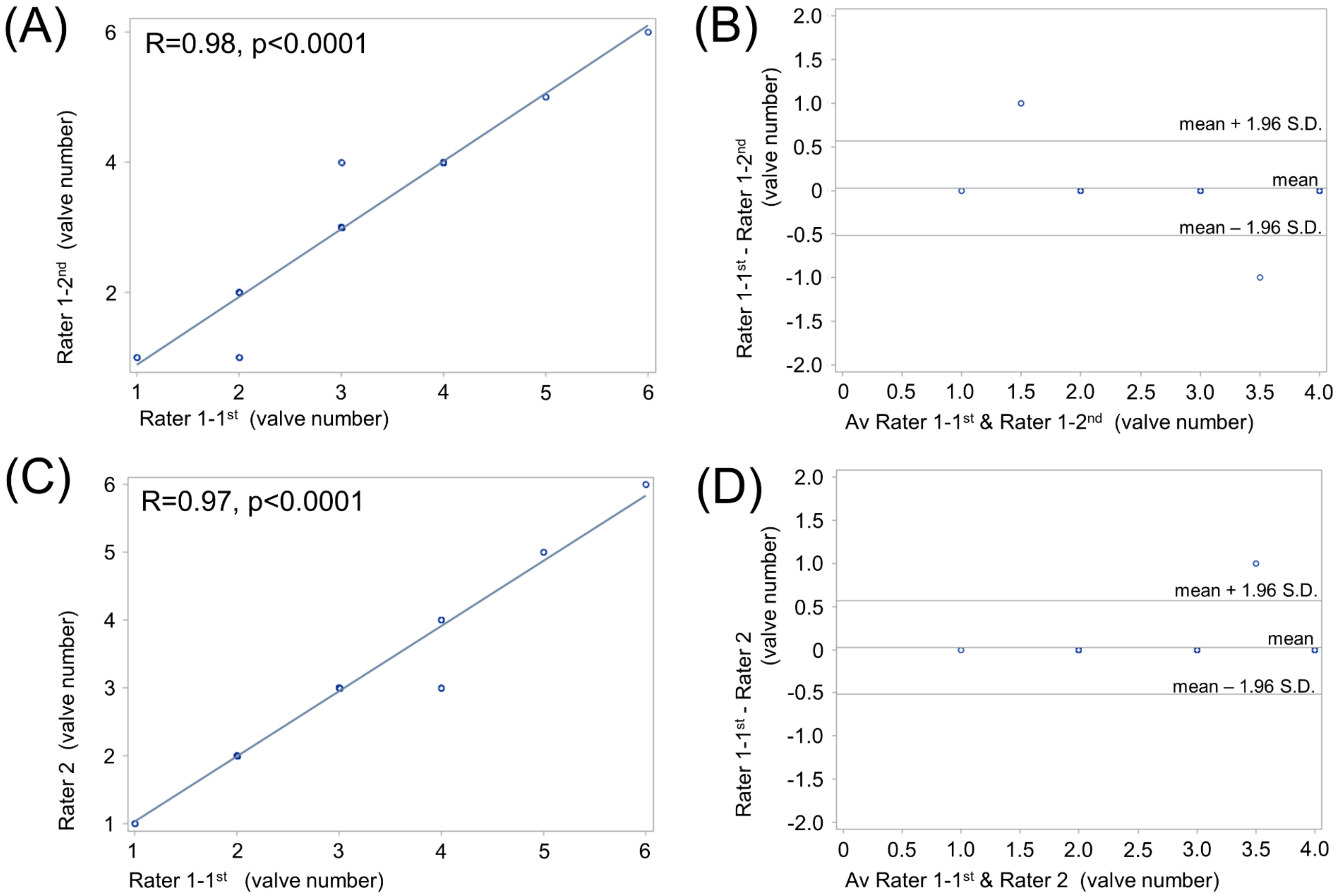

Supplemental Figure.3

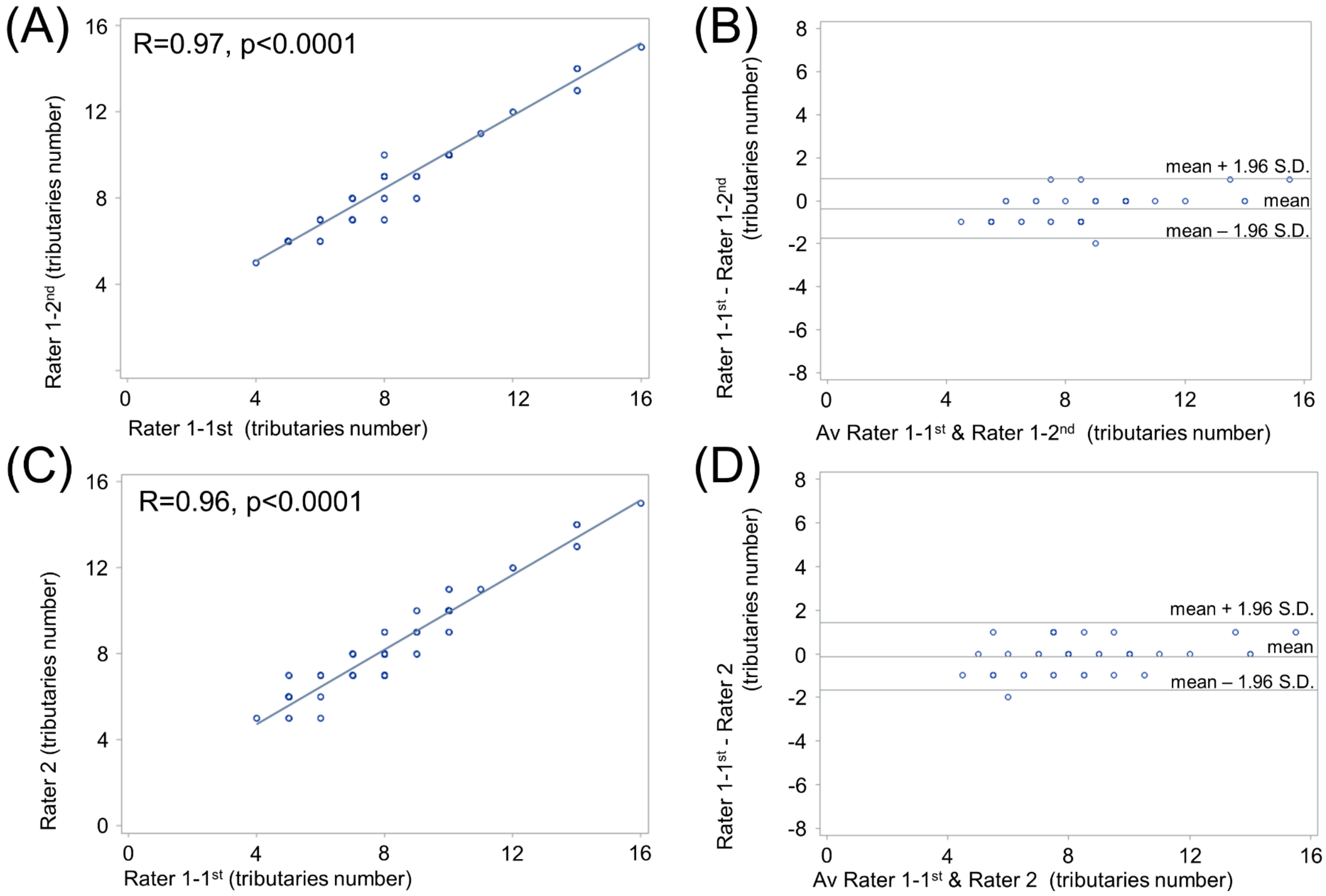

Supplemental Figure.4

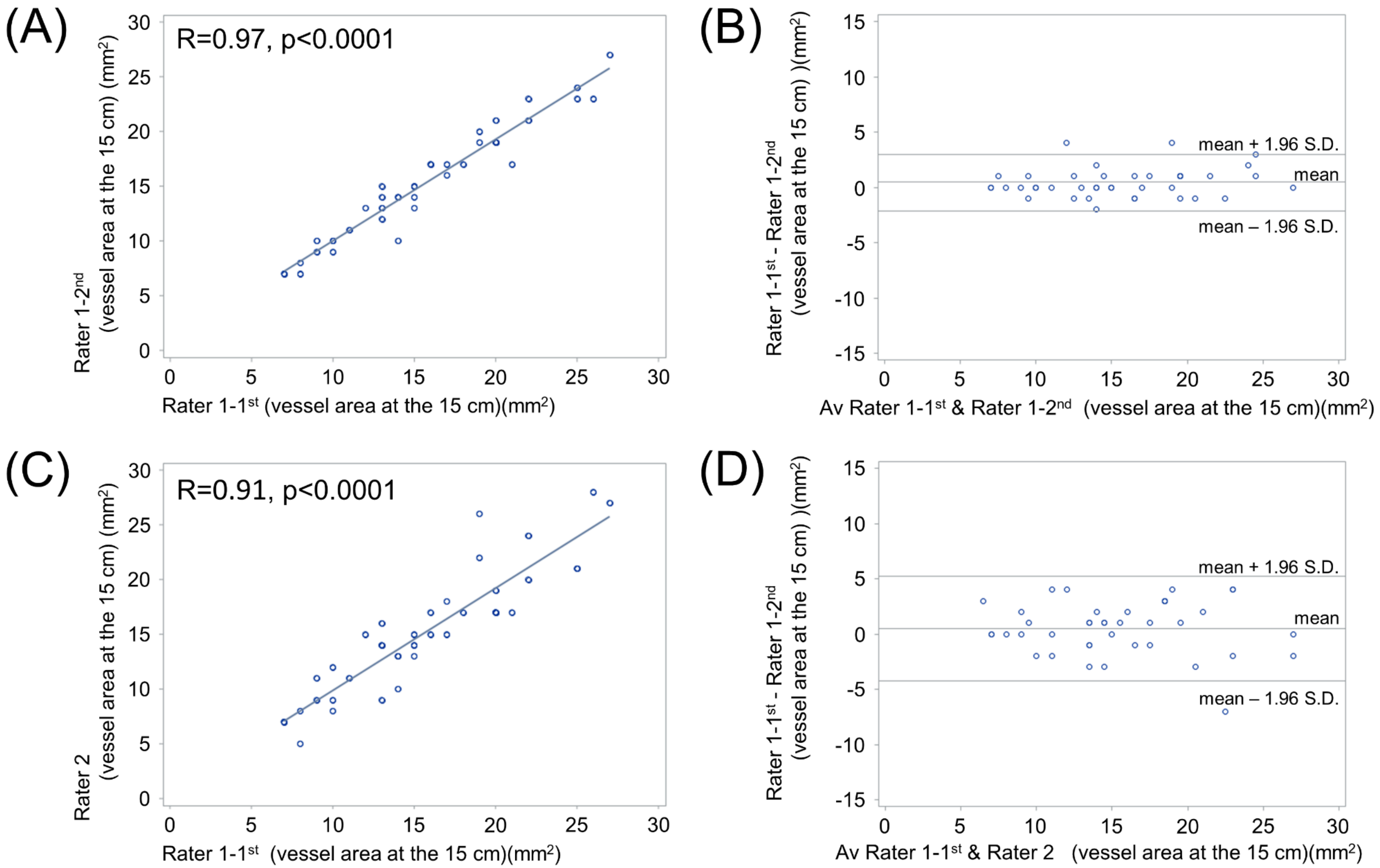

Supplement: Supplementary file 1 — Supplementary Figures. [file 41598_2021_90998_MOESM1_ESM.pdf]
